# Multimodal ultrasound-based morphological differences between symptomatic and asymptomatic carotid web

**DOI:** 10.3389/fneur.2026.1664894

**Published:** 2026-06-24

**Authors:** Chenyang Dai, Shihao Ruan, Linlin Li, Yuanyuan Tang, Lu Wang, Kai Wang

**Affiliations:** 1 Department of Neurology, The First Affiliated Hospital of Anhui Medical University, Hefei, China; 2 Anhui Province Key Laboratory of Cognition and Neuropsychiatric Disorders, Hefei, China; 3 Collaborative Innovation Center of Neuropsychiatric Disorders and Mental Health, Hefei, Anhui, China; 4 Anhui Provincial Institute of Translational Medicine, Anhui Medical University, Hefei, China; 5 The School of Mental Health and Psychological Sciences, Anhui Medical University, Hefei, China

**Keywords:** carotid web, computed tomography angiography, digital subtraction angiography, ischemic stroke, multimodal ultrasound

## Abstract

**Objective:**

We aimed to investigate the differences between symptomatic and asymptomatic carotid webs (CWs) using clinical and ultrasound morphological features and identify potential risk factors for CW-associated ischemic stroke.

**Methods:**

We retrospectively recruited 45 patients diagnosed with CW between January 2021 and September 2024 using digital subtraction angiography or computed tomography angiography. Patients were categorized according to the occurrence of CW-associated stroke. Multimodal ultrasound was used to identify the morphological characteristics of the CWs, carotid stenosis rate and hemodynamic alterations. Binary-variable logistic regression was used to assess the high-risk ultrasound morphologic features of CW-induced ischemic stroke.

**Results:**

Forty-five patients with CWs were identified, of whom 15 had stroke-related symptomatic CWs. Symptomatic patients demonstrated higher hyperlipidemia prevalence, had greater web dimensions (length, 5.80 ± 1.64 vs. 4.41 ± 1.67 mm, *p* = 0.011; thickness, 1.88 ± 0.43 vs. 1.38 ± 0.39 mm, *p* < 0.001), and a higher prevalence of curved CW shape (80.0% vs. 43.3%, *p* = 0.044). CW-induced stenosis was more severe in the symptomatic group than in the asymptomatic group (46.35 ± 9.89% vs. 40.13 ± 11.44%), although the majority of the stenoses were mild (<50%). Hemodynamically, while flow velocities were comparable, symptomatic cases showed significantly lower resistance index (0.59 ± 0.10 vs. 0.65 ± 0.07, *p* = 0.02). Multivariate analysis identified CW length (OR = 2.10, 95% CI 1.08–4.06; *p* = 0.028), CW thickness (OR = 21.80, 95% CI 2.43–196.00; *p* = 0.006), and lower RI as independently associated factors of symptomatic CWs. ROC analysis yielded optimal cutoffs of >1.65 mm for thickness (AUC = 0.813), > 5.20 mm for length (AUC = 0.723), and ≤ 0.691 for RI (AUC = 0.698).

**Conclusion:**

Symptomatic CWs exhibit distinct morphological and hemodynamic features than asymptomatic CWs. These ultrasound-detectable characteristics may help identify high-risk patients for stroke prevention.

## 1 Introduction

Stroke remains a leading cause of death and disability worldwide, with ischemic stroke constituting the majority of cases (1). According to epidemiological data, approximately 2.4 million new stroke cases occur annually in China, and the burden of ischemic stroke is particularly severe, contributing significantly to morbidity, mortality, and healthcare expenditures (2). Cryptogenic stroke, wherein no definitive cause is identified despite thorough evaluation, accounts for approximately 25–30% of all ischemic strokes (3, 4). Emerging evidence suggests carotid webs (CWs) may represent an underrecognized etiology, especially in younger patients lacking traditional cardiovascular risk factors (5). Studies indicate CWs are present in up to 13% of patients with cryptogenic stroke aged <60 years, with alarmingly high recurrence rates of 11.4–27.3% despite medical therapy (6–8). This underscores the critical need for early detection and intervention to mitigate stroke risk.

CWs are characterized by shelf-like projections in the lumen and are typically located in the proximal posterior wall of the internal carotid artery (ICA). Histologically distinct from atherosclerotic plaque, CWs consist of fibromuscular dysplasia-like tissue believed to result from abnormal intimal hyperplasia. The pathophysiological mechanism involves interruption and stagnation of blood flow distal to the web, creating a thrombogenic environment that can lead to artery-to-artery embolism and subsequent ischemic stroke (9). Current diagnostic approaches include ultrasound as a first-line non-invasive screening tool, complemented by computed tomography angiography (CTA) or magnetic resonance angiography (MRA) for improved detection, with digital subtraction angiography (DSA) remaining the gold standard for definitive diagnosis. Current AHA guidelines recommend noninvasive carotid imaging within 48 h for eligible TIA or minor stroke patients (10). Evidence from De Athayde Soares et al. (11) has confirmed equivalent post-procedural outcomes in stroke incidence, survival, and vascular patency between the carotid ultrasound-alone group and the combined CTA/MRA group, which demonstrates the reliability of ultrasound as a single pre-procedural assessment tool for lesions including CW. However, ultrasound remains underutilized due to variable sensitivity and the absence of standardized criteria to distinguish symptomatic from asymptomatic CWs. Multimodal ultrasound technology integrates various sonographic techniques, providing more comprehensive information for the diagnosis of CWs (12). Common gray-scale imaging can visualize the characteristic thin, linear membrane protruding into the lumen, aiding in its differentiation from other vascular abnormalities. However, its diagnostic validity is limited, particularly in the axial plane, and requires meticulous scanning techniques by an experienced operator to achieve reliable identification, as its sensitivity is not universal (13). Color Doppler flow imaging (CDFI) reveals important hemodynamic information by detecting flow turbulence or stasis distal to the web, while spectral Doppler quantifies velocity changes indicative of flow obstruction. B-flow imaging offers unique advantages by directly visualizing blood flow without Doppler-related artifacts. This comprehensive approach combines morphological assessment with functional hemodynamic evaluation, potentially improving the prediction of stroke risk associated with CWs.

The clinical significance of CW-associated stroke deserves to be emphasized, particularly given the high recurrence rates reported (7). This highlights the urgent need for improved diagnostic accuracy and the development of reliable ultrasound criteria to identify high-risk CWs. Current literature lacks reliable evidence correlating specific sonographic features with ipsilateral stroke occurrence (12, 14). Addressing this knowledge gap could have a significant impact on clinical decision-making and may guide the choice of treatment options, such as conservative medication or surgical interventions like stenting or endarterectomy.

This study systematically investigated the distinctive ultrasound characteristics of symptomatic CWs through comprehensive morphological and hemodynamic features of symptomatic versus asymptomatic cases. Additionally, this study aims to enhance clinicians’ understanding of the mechanisms underlying CW-related strokes and promote more timely and appropriate intervention measures, ultimately reducing the incidence of strokes in this underestimated patient population.

## 2 Methods

### 2.1 Participants

This retrospective study screened the institutional database of the First Affiliated Hospital of Anhui Medical University (January 2021–September 2024) for patients with suspected or reported carotid webs (CW) on CTA or DSA. Initial screening identified 57 patients. Their original CTA/DSA images were reviewed to confirm a typical CW diagnosis. Subsequently, patients were excluded for: (1) poor-quality carotid ultrasound (*n* = 4); (2) unavailability of original CTA/DSA for review (*n* = 3); or (3) insufficient clinical history (*n* = 5). The final cohort consisted of 45 patients with confirmed CW and complete data. Their carotid ultrasound images were retrospectively analyzed for morphological and hemodynamic assessment. The study was approved by the Ethics Committee of Anhui Medical University (2021H048), and informed consent was obtained from all participants.

The eligible patients were divided into symptomatic and asymptomatic groups. Symptomatic patients were included only after a comprehensive evaluation confirmed the CW as the most probable cause of their ischemic event. This determination required the concurrent satisfaction of three criteria: (1) radiographic confirmation of acute infarction in the ipsilateral anterior circulation territory for stroke, or typical transient neurological deficits for TIA; (2) confirmation of a typical CW in the ipsilateral carotid bulb on the CTA or DSA study used for enrollment; and (3) exclusion of alternative major stroke mechanisms, including atrial fibrillation or other high-risk cardiac embolic sources, significant atherosclerotic stenosis (>50%) outside the CW location, intracranial large artery stenosis ≥50%, or other definite causes. For these symptomatic patients, the carotid ultrasound examination was performed in the subacute phase, typically 2 to 4 weeks after the index event, following clinical stabilization. The asymptomatic group included patients with no history of ipsilateral ischemic events, regardless of comorbid conditions. The asymptomatic group consisted of patients with no history of ipsilateral ischemic stroke or TIA, who underwent carotid imaging for the following indications: (1) screening for cardiocerebrovascular risk factors (e.g., hypertension, diabetes, hyperlipidemia); (2) evaluation of non-specific neurological symptoms such as dizziness, chronic headache, or vertigo; and (3) incidental discovery during work-up for contralateral cerebrovascular events or other head and neck conditions.

### 2.2 Clinical data collection and follow-up

Baseline characteristics and relevant risk factors were collected, including age, sex, hypertension, diabetes, hyperlipidemia, smoking history, alcohol use, atrial fibrillation, clinical symptoms, and clinical management. Follow-up interviews were conducted by reviewing electronic medical records or telephone interviews of the patients. The primary endpoint was cerebrovascular event occurrence. Follow-up data regarding endpoint events were obtained by reviewing electronic medical records and, when necessary, supplemented by structured telephone interviews. For each patient, the follow-up period was defined as the time from the baseline carotid ultrasound examination to the last documented clinical encounter or the occurrence of a cerebrovascular event (the primary endpoint).

### 2.3 Carotid ultrasound protocol

**Figure 1:**
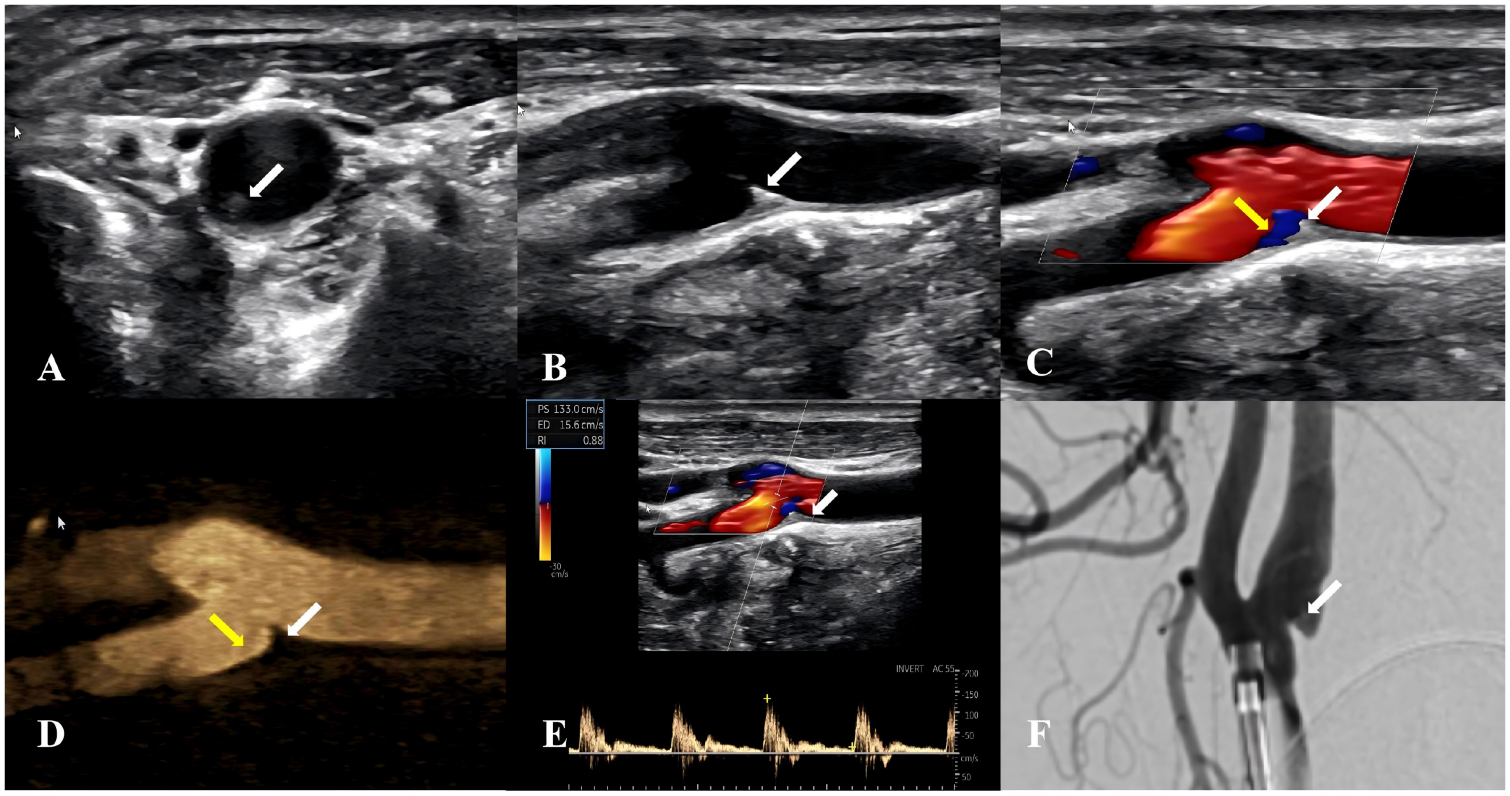
Multimodal ultrasound image of carotid web (white arrows) located in the posterior wall of the right carotid artery at its origin. **(A)** An isoechoic membrane-like structure is shown in the transverse ultrasound image. **(B)** A short, thin, and linear bilinear structure in longitudinal ultrasound images. **(C)** Color Doppler flow imaging revealed swirling flow (yellow arrow) at the base of the carotid web. **(D)** B-flow imaging delineates the carotid web with high clarity without color Doppler’s overflow artifacts, simultaneously revealing the morphology and localized blood flow disturbances (yellow arrow). **(E)** Spectral Doppler measurements at the carotid web. **(F)** Corresponding digital subtraction angiography (DSA) image shows the definitive linear filling defect.

Experienced neurovascular ultrasonographers conducted carotid ultrasound examinations using a LOGIQ E11 (GE Healthcare, Chicago, IL, USA) ultrasound system equipped with a 3–12 MHz linear transducer. Patients who underwent carotid ultrasound were first subjected to scanning using the probe from the clavicle to the submandibular region in a continuous transverse view, followed by a longitudinal view. The scans were conducted and archived with the knowledge of the established CW diagnosis from prior CTA/DSA. Once CW was detected on scanning, Upon detection of a CW, its morphological characteristics were assessed using a multimodal ultrasound protocol, which included: gray-scale imaging in transverse (Figure 1A) and longitudinal planes (Figure 1B), color flow imaging (Figure 1C), B-flow mode (Figure 1D), and spectral Doppler (Figure 1E), with the corresponding diagnostic DSA shown in Figure 1F. Multiple cine loops and still images were stored to facilitate the subsequent retrospective identification of the clearest frames for precise measurement.

### 2.4 Carotid ultrasound analysis

**Figure 2:**
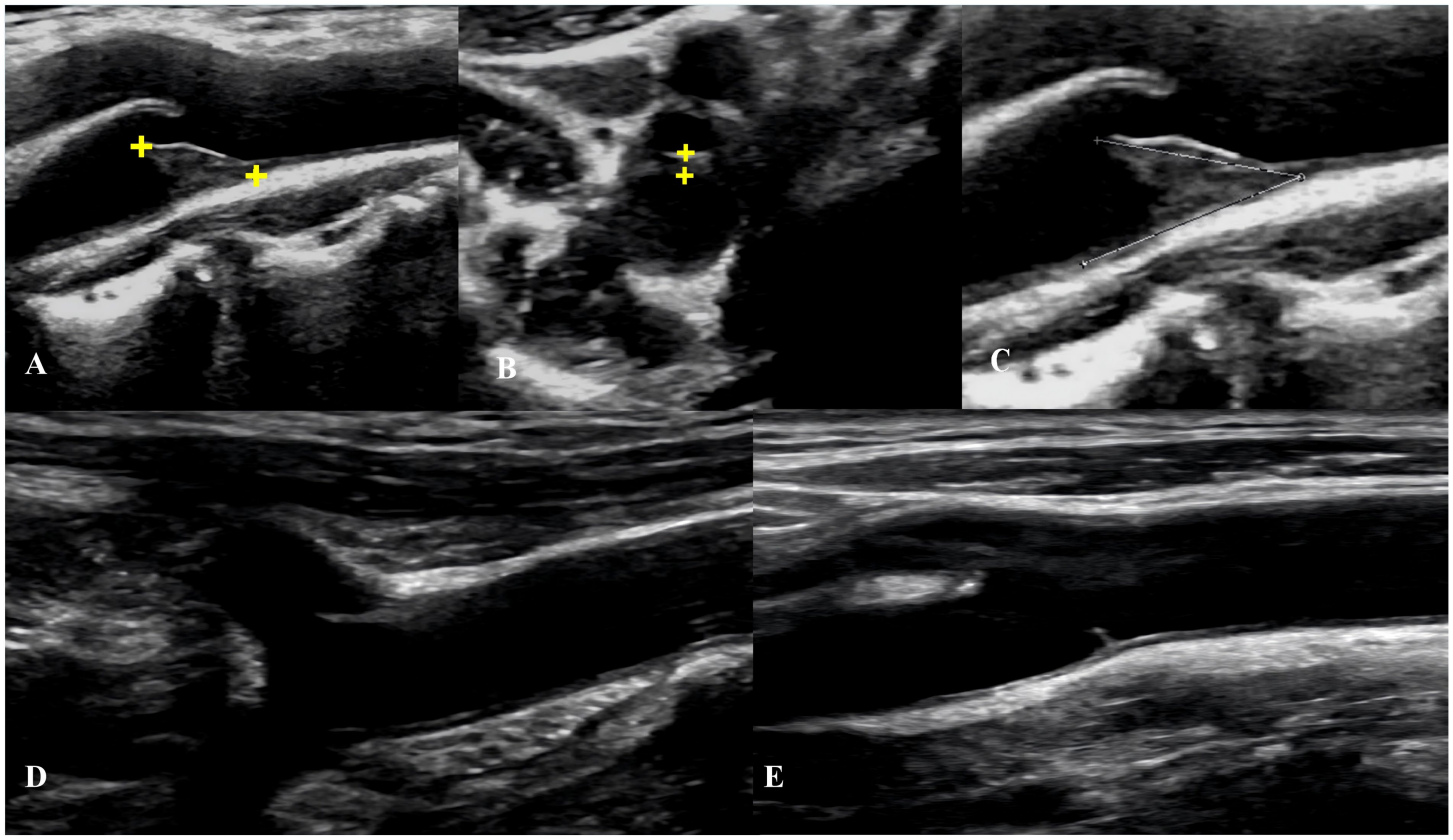
Measurements and morphological types of carotid web on ultrasound images. **(A)** Measure the length of the carotid web on the longitudinal section, measuring the distance between the tip of the carotid web and the origin of the base of the carotid web. **(B)** Measure the thickness of the carotid web at a point where the thickness is relatively uniform on the cross-section. **(C)** Measurement of carotid web angle using Adobe Photoshop CS3. **(D)** Representative longitudinal B-mode image of a curved CW. **(E)** Representative longitudinal B-mode image of a straight CW.

All observations were reviewed by two experienced diagnostic sonographers and interpreted by consensus. For this retrospective analysis, the two sonographers performing the measurements were blinded to the patients’ clinical symptomatic status. To ensure reproducibility, scanning was standardized: the longitudinal plane was adjusted to clearly delineate the anterior and posterior vessel walls for length and shape assessment; the cross-sectional plane was adjusted to obtain a perpendicular view through the web for thickness measurement. On common gray-scale imaging, the following CW parameters were assessed: side (left or right artery); location (anterior, posterior, or side arterial wall; defined on cross-sectional imaging: anterior wall facing the patient’s anterior direction, posterior wall facing posteriorly, lateral wall facing laterally relative to the carotid bulb anatomy); length; thickness; shape (straight: linear or planar superior border maintaining a consistent angle to the vessel wall; or curved: distinct convex superior border arching into the arterial lumen, assessed in the longitudinal B-mode plane) with representative examples provided in Figure 2D (curved) and Figure 2E (straight); and co-existent atherosclerotic plaque size at the CW base, if present. Atherosclerotic plaque was defined as inner-medial membrane thickening greater than 1.5 mm or localized thickening exceeding half of the surrounding tissue. The measurement protocol for CW length was to clearly display the largest protruding portion of the CW in the carotid ultrasound longitudinal section, and the distance from the origin of the base of the CW to the farthest distance from the tip was measured (Figure 2A). The CW thickness was measured in the cross-sectional plane. The probe angle was adjusted to make the diaphragm-like structure perpendicular to the ultrasound beam. To avoid the wider base, thickness was measured at the mid-portion of the CW body, where the structure is relatively uniform, ensuring the measurement line was perpendicular to the plane of the CW (Figure 2B). Both length and thickness measurements were repeated 3 times and averaged (in mm, accurate to 0.1 mm). CDFI and B-flow patterns were used to detect whether CW resulted in regional blood flow disturbances, such as turbulent and swirling flows. According to the North American Symptomatic Carotid Endarterectomy Trial criteria, multimodal ultrasound was used to evaluate the degree of carotid stenosis caused by CW, and the degree of stenosis at the CW was categorized as <50% and ≥50% stenosis. Spectral Doppler measurement was performed at CW, and the peak systolic velocity (PSV) and end-diastolic velocity (EDV) of the carotid artery were recorded. The resistance index (RI) was derived automatically using the formula: RI = (PSV − EDV)/PSV. The CW angle is the acute angle between the straight line connecting the top of the CW to its root and the carotid artery wall. CW angles were measured using the open-source computer software Adobe Photoshop CS3 (Adobe, San Jose, CA, USA) (Figure 2C).

### 2.5 Statistical analysis

The measurement data conforming to a normal distribution are presented as mean ± standard deviation. Categorical variables are presented as percentages. Differences between groups were compared using an independent sample *t*-test, chi-square test, or Fisher’s exact test. To test for consistency between observers in the measurement of the CW angle, the same author reanalyzed all cases after an interval of 1 month. Significance was set at *p* < 0.05, and all *p* values were two-sided. To control for the increased risk of type I error due to multiple comparisons, a Benjamini-Hochberg false discovery rate (FDR) correction was applied to the between-group comparisons of the key sonographic variables presented in Table 1. Binary logistic regression was performed to identify independently associated factors of symptomatic status. Variables showing significant univariate associations (*p* < 0.05) were considered for multivariate modeling, including hyperlipidemia, CW length, thickness, shape, stenosis rate, and resistance index. The RI was standardized (converted to z-scores) prior to inclusion in a forward stepwise logistic regression model with entry criteria of *p* < 0.05. We used binary logistic regression to estimate the odds ratio (OR) between CW and stroke, along with its 95% confidence interval (CI). Receiver operating characteristic (ROC) curve analysis was employed to evaluate the diagnostic performance of the independently associated factors identified in the final regression model, with optimal cutoff values determined by maximizing Youden’s index. Pearson’s correlation analysis was performed using the IBM SPSS Statistics 24 (IBM, Armonk, NY, USA).

**Table 1:** Comparison of morphological characteristics in ultrasound.

Variable	Symptomatic CW (*n* = 15)	Asymptomatic CW (*n* = 30)	*t*/*χ*^2^	*p* value
Side, *n* (%)			0.045	0.832
Left	7 (46.7%)	13 (43.3%)		
Right	8 (53.3%)	17 (56.7%)		
Location, *n* (%)			4.179	0.092
Anterior	6 (40.0%)	5 (16.7%)		
Posterior	7 (46.7%)	23 (76.7%)		
Lateral	2 (13.3%)	2 (6.7%)		
CW length (mm)	5.80 ± 1.64	4.41 ± 1.67	−2.519	0.016*
CW thickness (mm)	1.88 ± 0.43	1.38 ± 0.39	−2.127	<0.001*
CW angle (°)	33.64 ± 21.77	43.09 ± 22.79	1.351	0.187
Shape, *n* (%)			4.061	0.044*
Curved	12 (80.0%)	13 (43.3%)		
Linear	3 (20.0%)	17 (56.7%)		
Echogenicity, *n* (%)			0.000	1.000
Isoechoic	10 (66.7%)	20 (66.7%)		
Hypoechoic	5 (33.3%)	10 (33.3%)		
Stenosis rate (%)	46.25 ± 9.89	40.13 ± 11.44	−2.097	0.042*
Stenosis degree, *n* (%)			0.963	0.464
<50%	10 (66.7%)	24 (80.0%)		
≥50%	5 (33.3%)	6 (20.0%)		
PSV	91.07 ± 31.09	92.93 ± 35.95	0.171	0.865
EDV	31.50 ± 12.38	35.53 ± 9.88	−1.183	0.245
RI	0.59 ± 0.10	0.65 ± 0.07	2.416	0.020*
CW with co-existent atherosclerotic plaque, *n* (%)	7 (46.7%)	18 (60.0%)	0.720	0.396
Co-existent plaque length (mm)	8.87 ± 4.95	7.90 ± 3.50	−0.754	0.459
Co-existent plaque thickness (mm)	2.61 ± 0.71	2.69 ± 1.16	0.018	0.985

Data are presented as mean ± standard deviation or number (percentage).

CW, Carotid web; PSV, Peak Systolic Velocity; EDV, End-Diastolic Velocity; R, Resistive Index.

*p < 0.05.

## 3 Results

### 3.1 Baseline characteristics of patients

A total of 45 patients with CW were included in the study following the application of the inclusion and exclusion criteria, including 15 symptomatic and 30 asymptomatic patients with CW (Table 2). The two groups showed no significant difference with respect to age (*p* = 0.133) and sex (*p* = 0.063). The proportion of patients with hyperlipidemia was significantly higher in the symptomatic group than in the asymptomatic group (60.0% vs. 16.7%, *p* = 0.003), while no significant differences were observed in other risk factors including hypertension, diabetes mellitus, smoking, alcohol use, or atrial fibrillation (all *p* > 0.05). Fifteen patients with symptomatic CW suffered ipsilateral anterior circulation ischemic events: 11 patients had a stroke, and 4 had a transient ischemic attack (TIA). Among the stroke patients, the majority (81.82%) were classified as having a mild stroke, defined as a National Institutes of Health Stroke Scale/Score (NIHSS) of ≤ 3 points. Of these patients, 86.67% had baseline modified Rankin Scale (mRS) score of 0–2. Thirty patients with asymptomatic CW were examined for headache, dizziness, stroke in other areas, or other reasons.

**Table 2:** Baseline characteristics.

Characteristic	Symptomatic CW (*n* = 15)	Asymptomatic CW(*n* = 30)	*t*/*χ*^2^	*p* value
Age (years)	60.87 ± 12.64	66.33 ± 10.58	1.530	0.133
Female, *n* (%)	7 (46.7%)	6 (20.0%)	3.462	0.063
Hypertension, *n* (%)	10 (66.7%)	15 (50.0%)	1.125	0.289
Diabetes mellitus, *n* (%)	3 (20.0%)	8 (26.7%)	0.015	0.902
Hyperlipidemia, *n* (%)	9 (60.0%)	5 (16.7%)	8.762	0.003
Smoking, *n* (%)	5 (33.3%)	5 (16.7%)	1.607	0.205
Alcohol Use, *n* (%)	2 (13.3%)	6 (20.0%)	0.019	0.890
Atrial fibrillation, *n* (%)	0 (0%)	2 (6.7%)	N/A	0.545

Categorical variables were compared using the *χ*^2^ test or Fisher’s exact test, as appropriate. The test statistic for Fisher’s exact test is denoted as N/A.

CW, Carotid web.

### 3.2 Clinical outcomes during follow-up

Among the 15 conservatively managed symptomatic patients with CW (follow-up range: 2–36 months), cerebrovascular events occurred in 3 cases (20%). Two of these patients subsequently underwent carotid artery stenting, with no further stroke recurrence post-intervention. Contrastingly, none of the 30 asymptomatic CW patients experienced ischemic stroke or TIA during extended surveillance (12–40 months), with all maintaining clinical stability throughout outpatient monitoring.

### 3.3 Morphological characteristics of CW on ultrasound

The morphological characteristics of the CWs are listed in Table 1. The CWs in the symptomatic group were relatively longer (5.80 vs. 4.41 mm, *p* = 0.011) and thicker (1.88 vs. 1.38 mm, *p* < 0.001). A curved shape was significantly more common in symptomatic CWs (80.0%) than in asymptomatic CWs (43.3%) (*p* = 0.044). The qualitative assessment of CW echogenicity (isoechoic vs. hypoechoic) did not differ between symptomatic and asymptomatic groups, with an identical prevalence of isoechoic CWs (66.7%) in both cohorts (*p* = 1.000). Despite a statistically higher degree of stenosis in the symptomatic group (46.25 ± 9.89% vs. 40.13 ± 11.44%, *p* = 0.042), the overwhelming majority of patients in both groups had only mild stenosis (< 50%), with no significant difference in its prevalence (66.7% vs. 80.0%, *p* = 0.464). Spectral Doppler imaging at the CW level showed no statistically significant differences in PSV (91.07 ± 31.09 vs. 92.93 ± 35.95 cm/s, *p* = 0.865) or EDV (31.50 ± 12.38 vs. 35.53 ± 9.88 cm/s, *p* = 0.245) between the symptomatic and asymptomatic CW groups. However, the RI was significantly lower in the symptomatic than the asymptomatic group (0.59 ± 0.10 vs. 0.65 ± 0.07, *p* = 0.020). The two groups did not differ regarding the CW side (left: 46.7% vs. 43.3%, *p* = 0.832), location (*p* = 0.092), or angle (33.64 ± 21.77° vs. 43.09 ± 22.79°, *p* = 0.187). Similarly, the presence of co-existent atherosclerotic plaque (46.7% vs. 60.0%, *p* = 0.396) and plaque size (length: 8.87 ± 4.95 vs. 7.90 ± 3.50 mm, *p* = 0.459; thickness: 2.61 ± 0.71 vs. 2.69 ± 1.16 mm, *p* = 0.985) were comparable between groups. After applying the Benjamini-Hochberg FDR correction (*α* = 0.05), the differences in CW thickness (corrected *p* = 0.005), length (corrected *p* = 0.028), and resistive index (corrected *p* = 0.033) remained statistically significant. The differences in stenosis rate and CW shape yielded corrected *p*-values of 0.044.

Inter-observer reliability analysis demonstrated excellent agreement for continuous measurements, with intraclass correlation coefficients (ICC) of 0.92 (95% CI: 0.87–0.96) for CW thickness and 0.89 (95% CI: 0.82–0.94) for CW length. For categorical assessment of CW shape, substantial agreement was achieved with a Cohen’s kappa of 0.82.

To assess the robustness of our findings, a sensitivity analysis was conducted after excluding the four patients with transient ischemic attack (TIA) from the symptomatic group. In this refined analysis comparing the stroke-only symptomatic group with the asymptomatic group, the key morphological differences remained robust: symptomatic CWs were significantly more likely to be longer (*p* = 0.034), thicker (*p* = 0.002), and curved (*p* < 0.030). Regarding hemodynamic parameters, the Resistive Index (RI) remained significantly lower in the symptomatic group (*p* = 0.012), while the difference in stenosis severity was attenuated and no longer statistically significant (*p* = 0.052).

### 3.4 Binary logistic regression of ultrasonic morphological characteristic in symptomatic and asymptomatic CWs

The forward stepwise regression model identified three independently associated factors of symptomatic CWs (Table 3). Hyperlipidemia, considered as a potential confounder, was not retained in the final model.

**Table 3:** Binary logistic regression analysis for Independently associated factors of symptomatic carotid webs.

Independently associated factor	Odds ratio	95% confidence interval	*p* value
CW thickness	21.80	2.43–196.00	0.006
CW length	2.10	1.08–4.06	0.028
Standardized RI	0.27	0.10–0.72	0.009

CW, Carotid web; OR, Odds Ratio; RI, Resistive Index.

### 3.5 Diagnostic performance and cutoff values of the independently associated factors from logistic regression

To translate the independently associated factors into clinically applicable tools, Receiver Operating Characteristic (ROC) curve analyses were performed. The area under the curve (AUC), optimal cutoff values, and corresponding sensitivity and specificity for predicting symptomatic status are summarized in Table 4. CW thickness demonstrated excellent discriminatory power (AUC = 0.813), with an optimal cutoff of > 1.65 mm providing high specificity (86.7%). CW length showed good discriminatory power (AUC = 0.723), with a cutoff of > 5.20 mm yielding balanced sensitivity (73.3%) and specificity (66.7%). The Resistance Index (RI) was a valuable inverse predictor; a cutoff of ≤ 0.691, indicating lower vascular resistance, was associated with symptomatic CWs with high sensitivity (86.7%) and acceptable specificity (70.0%). These cut-off values and their performance metrics were derived from the present cohort; external validation is required to confirm their generalizability.

**Table 4:** Diagnostic performance of Independently associated factor for symptomatic carotid webs.

Independently associated factor	AUC (95% CI)	Optimal cutoff	Sensitivity (%)	Specificity (%)
CW thickness	0.813 (0.688–0.939)	> 1.65 mm	60.0	86.7
CW length	0.723 (0.570–0.876)	> 5.20 mm	73.3	66.7
RI	0.698 (0.519–0.877)	≤ 0.691	86.7	70.0

CW, Carotid web; RI, Resistive Index.

The symbols “>“and “≤” denote the value range associated with a higher risk of being symptomatic.

### 3.6 Correlation between carotid stenosis rate and CW ultrasonic morphological characteristics

**Figure 3:**
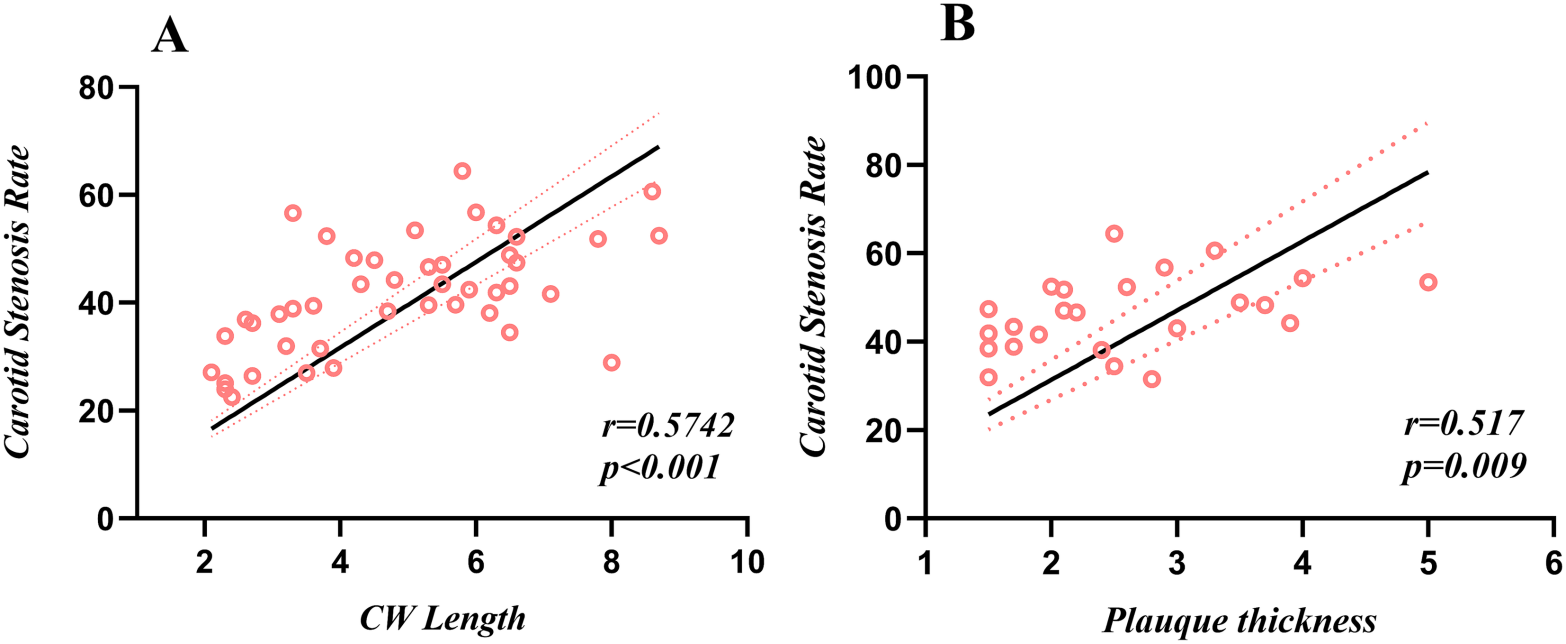
Correlation analysis between the carotid stenosis rate, carotid web length, and plaque thickness combined with carotid web. **(A)** Correlation analysis between carotid stenosis rate and carotid web length. **(B)** Correlation analysis between carotid stenosis rate and plaque thickness combined with carotid web.

Pearson’s correlation analysis suggested a positive correlation between the carotid stenosis rate and CW length (*r* = 0.601, *p* < 0.001), CW thickness (*r* = 0.431, *p* = 0.003), and a combined plaque thickness (*r* = 0.426, *p* = 0.034) (Figure 3).

## 4 Discussion

In this cross-sectional study, we investigated the morphological ultrasonic characteristics of symptomatic and asymptomatic CWs that induced stroke. The morphology of the ultrasound suggested that patients with a long, thick, and curved CW shape were at risk for acute ischemic stroke. The robustness of these key morphological findings was further confirmed by a sensitivity analysis excluding patients whose sole qualifying event was a transient ischemic attack. Furthermore, the symptomatic CW group exhibited significantly lower RI values than the asymptomatic CW group. This difference suggests that RI values may serve as a potential hemodynamic marker for assessing stroke risk in patients with CW. Notably, the significant difference in RI also persisted in our sensitivity analysis, reinforcing its potential role in risk stratification. These findings confirm that the primary mechanism is thromboembolism originating from the CW itself, not hemodynamic impairment.

CW is traditionally characterized as an atypical focal fibromuscular dysplasia of the carotid bulb that can potentially induce ipsilateral ischemic stroke (15, 16). Current research indicates that the overall incidence of CW is low, and large-scale epidemiological studies on the incidence of CW are currently lacking (17, 18). Advances in multiple head and neck vascular imaging techniques have increased awareness of CW as a contributing factor toward ischemic stroke. The pathogenesis remains unclear; however, it has been hypothesized that CW represents a congenital anomaly potentially attributable to genetic factors, vascular injury, or aberrant hormone levels within the body. CW is more prevalent in women than men, with an ethnic correlation (19). Several findings suggest that CW disturbs the balance of the intraluminal flow, thus causing the development of thrombosis and thrombus fragmentation, eventually leading to ischemic stroke (9). CW poses a significant risk of recurrent stroke in young patients without conventional stroke risk factors, although it is uncommon (20). This risk is compounded by the fact that delay in diagnosis remains common, as highlighted in a recent multicenter study which confirmed that such delays are independently associated with an increased risk of stroke recurrence (21). This evidence collectively underscores the need for heightened awareness and prompt recognition of this pathology.

These structural characteristics may contribute to stroke pathogenesis through several interrelated hemodynamic and thromboembolic mechanisms. First, the elongated and thickened morphology of CWs likely disrupts laminar blood flow, creating localized turbulence and flow stagnation near the web. This hemodynamic disturbance promotes endothelial injury and platelet activation, fostering thrombus formation. Second, the curvature of the web may further exacerbate flow separation and recirculation zones, increasing residence time for circulating platelets and coagulation factors, thereby enhancing thrombogenicity. These results are strongly supported by a recent large, multicenter CTA analysis by Bala et al. (22), which identified identical morphological traits, namely longer length and greater thickness, as independently associated factors of symptomatic CWs. This convergence of evidence across different imaging modalities underscores that these specific anatomical characteristics are critical for stroke risk stratification.

Notably, in our cohort, qualitative CW echogenicity was not associated with symptomatic status, suggesting that the intrinsic fibrotic composition may be a common feature, while the key determinant of symptom development appears to be the morphological characteristics that significantly alter local hemodynamics. This observation underscores the primacy of specific morphological features over echogenic appearance in determining stroke risk. The thin hyperechoic inferior edge, initially described by Fontaine et al. (13), was not consistently observed in our cohort, a finding consistent with other studies that emphasize the core shelf-like morphology without uniformly reporting this hyperechoic border (12, 14). Thus, its presence may be variable, influenced by technical factors or intrinsic tissue differences. Regarding the CW angle, our study found no significant difference between symptomatic and asymptomatic groups, contrasting with some CTA-based reports (22, 23). This discrepancy may be attributed to several factors. First, measurement methodology lacks standardization across studies; CTA-based approaches themselves use varying reference points, whereas we measured the angle at the CW’s proximal origin relative to the arterial wall to ensure ultrasonographic reproducibility. Additionally, our sample size may have been insufficient to detect subtle differences, and true biological variation across populations may exist. Critically, in our multivariate analysis, CW length and thickness emerged as stronger independently associated factors, suggesting these particular morphological parameters may be more dominant determinants of stroke risk in our cohort. Although hyperlipidemia was more prevalent in the symptomatic group, its lack of significance in our multivariate analysis suggests it is unlikely to be a meaningful confounder. This is consistent with the distinct non-atherosclerotic pathology of CWs, where thrombogenic mechanisms related to CW morphology rather than traditional vascular risk factors appear to drive stroke risk.

Most CW-induced carotid stenoses are mild (< 50%). Furthermore, the presence of even moderate-to-severe stenosis was not associated with symptomatic status, indicating that hemodynamic impairment is not the principal stroke mechanism. These findings, which align with previous studies, confirm that the primary mechanism is thromboembolism originating from the CW itself. The specific morphology of the CW fosters a pro-thrombotic environment, making this embolic potential the key determinant of clinical symptoms (24, 25). The symptomatic CW group exhibited significantly lower RI values than the asymptomatic CW group, although there were no significant differences in peak systolic and end-diastolic flow velocities between the two groups. This may be related to the following pathophysiological mechanisms: CW-induced hemodynamic disturbances may lead to distal microcirculatory embolism or reduced vascular bed resistance. The decreased RI values suggest compensatory dilation of distal vessels to maintain blood flow perfusion; in contrast, the RI values in the asymptomatic group remained relatively normal, indicating better hemodynamic compensation and the absence of significant microembolism or vascular regulatory abnormalities (26, 27). This difference suggests that RI values may serve as a potential hemodynamic marker for assessing stroke risk in patients with CW. It should be noted, however, that the difference in stenosis severity, while present in the primary analysis, was attenuated in the sensitivity analysis. This suggests that the relationship between stenosis severity and symptomatic status may be less robust than the morphological parameters, potentially due to reduced statistical power in the refined cohort.

With increasing recognition by diagnosticians, the imaging modalities currently available for CW diagnosis are DSA, CTA, and ultrasound. DSA is considered the gold standard imaging modality for CW diagnosis with reliability and safety; however, it remains an invasive technique. CW can be misdiagnosed if the DSA obtains only frontal and lateral standardized projections owing to the CW being mainly located in the posterior wall at the origin of the ICA, which has a posterior-lateral course. Carotid CTA has the advantages of high resolution, short scanning time, and the ability to construct images in any direction. Since CTA lacks dynamic visualization and no hemodynamic information is available, CW must be carefully differentiated from atherosclerotic plaque and dissection in diagnosis to prevent misdiagnosis (4, 16)^.^ Ultrasound, as a non-invasive and readily available tool, can provide valuable morphological and hemodynamic information for the assessment of CW in patients with an established diagnosis or high clinical suspicion. The advantages of ultrasound imaging are that it is radiation-free, inexpensive, has bedside maneuverability, and allows repeated dynamic observation. Ultrasound results depend heavily on the sonographer’s level of ultrasound technique and CW knowledge. Due to the paucity of reports on its characteristics, sonographers’ unfamiliarity with the ultrasound features of CW often leads to omission or misdiagnosis. Using a probe for multi-angle scanning during image acquisition provides access to clear CW images in both horizontal and longitudinal planes (13). Color Doppler enables real-time monitoring of swirling blood flow and sludging along the surface, which may cause thrombosis. B-flow is a technique that enhances the weak reflective signals of red blood cells and obtains ultrasound images similar to contrast-enhanced ultrasound images with high time-spatial resolution. B-flow is instrumental in detecting ICA stenosis by reducing flow artifacts within the stenosis and helping to characterize the CW morphology. This technique is particularly useful for characterizing CW morphology and detecting internal carotid artery stenosis by minimizing in-stenosis flow artifacts. Although B-flow provided excellent morphological delineation in this study, its reliance on vendor-specific technology may limit widespread applicability. Future studies should explore more accessible alternatives such as contrast-enhanced ultrasound (CEUS), which shows particular promise in directly visualizing the downstream thrombogenic flow disturbances critical to CW-related ischemic events (28, 29). Dynamic observation while scanning the carotid bulb and capturing movie images for review helps to monitor the presence of plaque or thrombosis at the basal part of the CW and to differentiate it from carotid limited dissection and ulcerated atherosclerotic plaques. Therefore, we retrospectively analyzed the morphological characteristics of ultrasound in patients diagnosed with CW by DSA or CTA. Both CTA and DSA are primarily defined by filling defects, whereas the morphological features of the CW itself can be directly observed and measured by ultrasonography intuitively and accurately.

In our study, we investigated patients with symptomatic and asymptomatic CWs. We identified stroke risk predictors based on morphological features detected by ultrasound to guide the subsequent treatment of CW patients. A relatively aggressive treatment regimen can be adopted for the high-risk group of CW to avoid the occurrence or recurrence of stroke. However, the optimal treatment for CW remains unclear. In this study, all symptomatic patients were initially managed medically. This reflects the real-world, retrospective nature of our study and the ongoing debate in our institution and the wider literature regarding the optimal management strategy for CW, particularly after a minor stroke or TIA. However, research suggests that oral medications alone for antithrombotic or antiplatelet therapy may not be sufficient to manage the incidence of stroke recurrence after the first exposure to CW, and approximately three patients (20%) in our study had stroke recurrence despite following a standardized pharmacological protocol. In the Haynes follow-up study, 52 patients with symptomatic CW had been treated with either dual antiplatelet and statin therapy or systemic anticoagulation, but all patients suffered ipsilateral recurrent stroke at a mean interval of 43 months (28). For symptomatic CWs, the two main surgical treatments are carotid endarterectomy (CEA) and carotid artery stenting (CAS), both of which require a careful individual risk–benefit assessment. Considering the safety of the procedure and its long-term efficacy in preventing stroke recurrence, CEA is the preferred treatment (30). In recent years, some investigators have also reported favorable results of revascularization using CAS for patients with CW (28, 31). In this study, two patients completed stenting and had no further stroke recurrence during follow-up (3–60 months). Therefore, considering the increased detection of CW in cryptogenic ischemic stroke, further evidence is required to determine the optimal therapeutic strategy for preventing stroke recurrence. Contrastingly, the optimal treatment for asymptomatic CW remains largely undetermined and is generally conservative.

Our study proposes preliminary cutoff values for CW thickness, length, and RI to aid in the risk stratification of CW patients. The discriminatory power of these parameters varied, with CW thickness (AUC = 0.813) being the strongest predictor, while the utility of RI (AUC = 0.698) was more modest. These findings suggest that morphological assessment is the cornerstone of ultrasound-based risk stratification, with RI providing supplementary hemodynamic information. The immediate clinical applicability of these thresholds, particularly RI, may be limited by their variable performance and need for standardized measurement. Nonetheless, this stratification holds significant potential for future clinical practice, as it may help identify patients who could benefit from more aggressive management.

## 5 Limitations

This study has some limitations. First, it was a retrospective, single-center study. The diagnosis of asymptomatic CW was based on the absence of clinical history, and their natural progression remains uncertain. Additionally, this design limited the systematic collection of certain anthropometric data, such as body mass index (BMI). The higher mean age in the asymptomatic cohort reflects the clinical indications for carotid imaging, which were predominantly screening for cardiocerebrovascular risk factors or evaluation of non-specific symptoms. This may influence the generalizability of our findings, although age did not differ significantly between groups (*P* = 0.133). Second, the statistical conclusions are constrained by the limited number of symptomatic events (*n* = 15). This small sample size increases the risk of overfitting and coefficient instability in the multivariable logistic regression model, which is directly reflected in the wide confidence intervals for some predictors (e.g., CW thickness). Therefore, the identified independently associated factors and their odds ratios should be considered exploratory. Moreover, the diagnostic cut-off values derived from this cohort are susceptible to optimism bias; their true clinical utility must be established through external validation in larger, prospective studies. Third, all patients with CW were identified using carotid CTA/DSA without histopathological evidence. Finally, an insufficient follow-up period may have underestimated the rate of stroke recurrence in some patients. Therefore, the definitive validation of these risk stratification criteria awaits larger, multi-center, prospective studies.

## 6 Conclusion

Multimodal ultrasound is a potent tool for the observation of the morphological features of CW. Our results suggest that there was a significant difference in ultrasound between CW in the symptomatic and asymptomatic groups, which is clinically relevant for the early identification of symptomatic CW and helpful for the prevention of stroke in high-risk patients.

## Data Availability

The raw data supporting the conclusions of this article will be made available by the authors, without undue reservation.
